# PelviSound L—Real‐time intraoperative sonographic assessment of pelvic sentinel lymph nodes using a drop‐in probe in endometrial and cervical cancer during minimally invasive surgery: A single‐center pilot feasibility study in 20 patients

**DOI:** 10.1002/ijgo.70838

**Published:** 2026-01-29

**Authors:** Sascha Hoffmann, Laura Trif, Bernhard Krämer, Felix Neis, Diethelm Wallwiener, Sara Y. Brucker, Markus Hoopmann, Markus Hahn

**Affiliations:** ^1^ Department of Women's Health University Women's Hospital Tübingen Tübingen Germany

**Keywords:** gynecologic cancer, image‐guided surgery, intraoperative ultrasonography, minimally invasive surgical procedure, sentinel lymph node biopsy

## Abstract

**Objective:**

Sentinel lymph node biopsy (SLNB) is considered an essential step in the surgical management of endometrial and cervical cancers as it has led to a reduction in the need for full pelvic lymphadenectomy. This proof‐of‐concept study aimed to evaluate the feasibility and diagnostic performance of intraoperative sonographic assessment of sentinel lymph nodes (SLNs) during laparoscopic and robotic surgeries for endometrial and cervical cancers.

**Methods:**

This was a prospective, single‐center, non‐interventional diagnostic feasibility study conducted between October 2023 and July 2024 at the University Women's Hospital, Tübingen, Germany, and registered at the German Clinical Trials Register (DRKS00032919). A total of 20 patients underwent intraoperative sonographic SLN evaluation using a sterile drop‐in ultrasound probe prior to resection. The assessment was based on the Vulvar International Tumor Analysis (VITA) criteria that includes parameters, such as lymph node shape, cortical thickening, vascularization, and echogenicity. Findings were correlated with histopathologic results to determine diagnostic accuracy. To assess the diagnostic accuracy of the sonographic criteria, a receiver operating characteristic (ROC) analysis was conducted, focusing on sensitivity and specificity with respect to lesion dignity classification (benign vs malignant).

**Results:**

Among the 25 initially prepped patients, 20 successfully underwent intraoperative sonographic SLN evaluation, while five were excluded due to logistical issues or consent withdrawal. A total of 49 SLNs were analyzed, and the analysis included histopathologic confirmation. The sonographic assessment demonstrated perfect sensitivity and specificity of 1.0 as reflected by the area under the ROC curve (AUC = 1.0). No adverse events were reported.

**Conclusion:**

The study confirms that intraoperative sonographic evaluation of SLNs in minimally invasive gynecologic oncology surgeries is feasible, safe, and highly accurate. This technique may serve as a valuable adjunct to current SLNB protocols and lead to a potential reduction in the need for extensive lymphadenectomy while concurrently maintaining diagnostic reliability. Further research using larger cohorts is warranted to validate these findings and assess broader clinical applicability.

## 1 INTRODUCTION

Sentinel lymph node biopsy (SLNB) is an established method used in the surgical treatment of endometrial and cervical cancers^1^ that has now made it possible to avoid a full pelvic lymphadenectomy if the sentinel lymph nodes (SLNs) are negative.^2^, ^3^ In this context, SLN mapping using indocyanine green (ICG) has recently proven to be a reliable method.^4^, ^5^ Minimally invasive laparoscopic or robotic surgical staging combined with SLNB now offers a low morbidity alternative to historical surgical management without compromising oncological outcomes.^6^ Ultrasound is an imaging modality for the evaluation of lymph nodes. It is increasingly being evaluated preoperatively^7^, ^8^, ^9^ for it accuracy and as a cost‐effective, readily available real‐time medium without radiation exposure; thus, it represents a promising option for intraoperative imaging.

The aim of this proof‐of‐concept‐study was to demonstrate that in situ sonographic assessment of SLN in laparoscopic and robotic‐assisted surgery for endometrial and cervical cancers is possible without causing patient‐related adverse effects and can be conducted without inducing patient‐related adverse effects and offers a reliable assessment of dignity.

## 2 MATERIALS AND METHODS

### 2.1 Study design

Between October 2023 and July 2024, we conducted a prospective, single center, non‐interventional diagnostic, cross‐sectional, proof‐of‐concept‐study PelviSound L in Germany (German Clinical Trials Register: https://drks.de/search/de/trial/DRKS00032919/details) to demonstrate the feasibility of intraoperative, in situ sonographic lymph node assessment. Ethical approval was obtained from an independent ethics committee (approval no. 315/2022B02). The study was conducted in full compliance with the Declaration of Helsinki and Good Clinical Practice (GCP) guidelines. Prior to study commencement, all participants provided written, informed consent.

The in situ evaluation of ICG‐positive SLNs was performed using intraoperative ultrasound prior to lymph node resection. A sterile drop‐in ultrasound probe that had been specifically designed for this purpose was introduced into the abdominal cavity through a 12‐mm trocar placed in the suprapubic region. Ultrasound imaging was performed using the bkActiv system (BK Medical, Denmark) equipped with the Rob12C4 transducer. The ultrasound assessment included a detailed sonographic examination of each lymph node using real‐time imaging documentation. Intraoperative ultrasound examination was performed by the operating surgeon directly during the surgical procedure. Following surgical exposure but prior to lymph node excision, the ultrasound probe was handled by the surgeon and applied to the target lymph node in real time. Sonographic assessment was conducted intraoperatively and immediately interpreted according to the predefined and standardized Vulvar International Tumor Analysis (VITA) criteria. The ultrasound findings were documented before lymph node removal. Although multiple surgeons (*n* = 3) were involved in the surgical procedures, intraoperative ultrasound interpretation and scoring were consistently performed by the study lead during surgery. This approach was chosen to reflect real‐world surgical conditions while ensuring standardized image interpretation and to evaluate the technical feasibility of surgeon‐performed intraoperative ultrasound assessment.

This approach was chosen to reflect real‐world surgical conditions and to evaluate the technical feasibility of surgeon‐performed intraoperative ultrasound assessment.

Morphological characteristics of the lymph nodes were analyzed using the VITA criteria that includes different parameters, such as lymph node size, shape, cortical thickening, vascularization, and echogenicity. These criteria were applied to systematically assess diagnostic status and establish a standardized evaluation framework. The findings were correlated with histopathologic results and preoperative imaging assessments to determine diagnostic accuracy.

Following completion of the sonographic evaluation and documentation, the ultrasound probe was removed, and resection of the sentinel lymph nodes was performed. Each resected sentinel lymph node sample was sent individually for histopathologic examination to establish a correlation with the ultrasound imaging findings.

### 2.2 Study outcomes

The primary outcome was the feasibility of using intraoperative sonography for the assessment of pelvic lymph nodes. Secondary endpoints were the evaluation of the sonographic assessment of individual lymph nodes based on the VITA criteria,^7^, ^10^ and the correlation of sonographic and VITA assessment with histopathologic findings.

### 2.3 Study population

The study included adult female patients who had been diagnosed with endometrial or cervical cancer and were referred for pelvic sentinel node biopsy or pelvic lymphadenectomy based on the multidisciplinary tumor board's recommendations. Eligible patients were required to be aged 18 years or older and have provided written informed consent after receiving detailed study information. Patients with known lymphadenopathy or anticipated compliance issues were excluded from participation.

### 2.4 Data collection

Patients' preoperative medical histories were recorded. Study data collection included intraoperative sonographic images and histopathologic findings which were digitally archived. Additionally, any adverse events in addition to duration of the procedure from the introduction of the ultrasound probe into the surgical site to its removal were documented.

### 2.5 Data analysis

Statistical analyses were performed using IBM SPSS Statistics for Windows (version 28.0.0.0; IBM Corporation, Armonk, New York, USA). The sample size calculation was based on an exact binomial test with a one‐sided significance level (alpha) of 0.05 and a statistical power of 92%. Under these conditions, it was determined that 20 patients would be sufficient to demonstrate the feasibility of intraoperative, in situ sonographic lymph node assessment.

An evaluation was done to determine whether intraoperative sonographic assessment of the sentinel node was feasible in all cases.

Assessments were performed based on the eight morphological VITA criteria in addition to the lymph node dimension in terms of long‐to‐short axis (L/S) ratio as shown in Table 1.

**TABLE 1:** Sonographic assessment criteria.

1. Nodal shape
2. Nodal‐core sign
3. Cortical thickening
4. Nodal echogenicity
5. Capsular interruption
6. Distortion of corticomedullary interface
7. Perinodal hyperechogenic ring
8. Grouping of lymph nodes (matting)
9. L/S ratio (lymph node dimension)

Abbreviation: L/S, long‐to‐short axis.

In terms of the L/S ratio, a value <2 was considered suspicious and assigned a score of one point. Similarly, the presence of abnormalities in any of the nine listed criteria was assigned one point. The cumulative score ranged from 0 to 9 and indicated the likelihood of lymph node metastasis with higher scores suggesting a greater probability of malignancy.

A receiver operating characteristic (ROC) curve analysis was used to evaluate the diagnostic performance of sonographic criteria. The area under the curve (AUC) was calculated to determine whether it could be applied to differentiate between benign and malignant lymph nodes.

Additionally, color and power Doppler assessments were conducted to evaluate blood‐vessel architecture and the color score. However, these parameters were not included in the calculation of the diagnostic score because the sample size was insufficient to derive statistically robust conclusions due to the complexity of the results.

In accordance with the journal's guidelines, we will provide our data for independent analysis by a selected team by the Editorial Team for the purposes of additional data analysis or for the reproducibility of this study in other centers if such is requested.

## 3 RESULTS

Out of 25 patients who were initially prepped for intraoperative ultrasounds, the procedure was successfully performed in 20 patients. Reasons for excluding the remaining patients included the unavailability of the ultrasound probe due to maintenance issues or use in other procedures (*n* = 3), lack of personnel to operate the ultrasound device (*n* = 1), or withdrawal of patient consent (*n* = 1).

### 3.1 Feasibility of sonographic assessment

**FIGURE 1:**
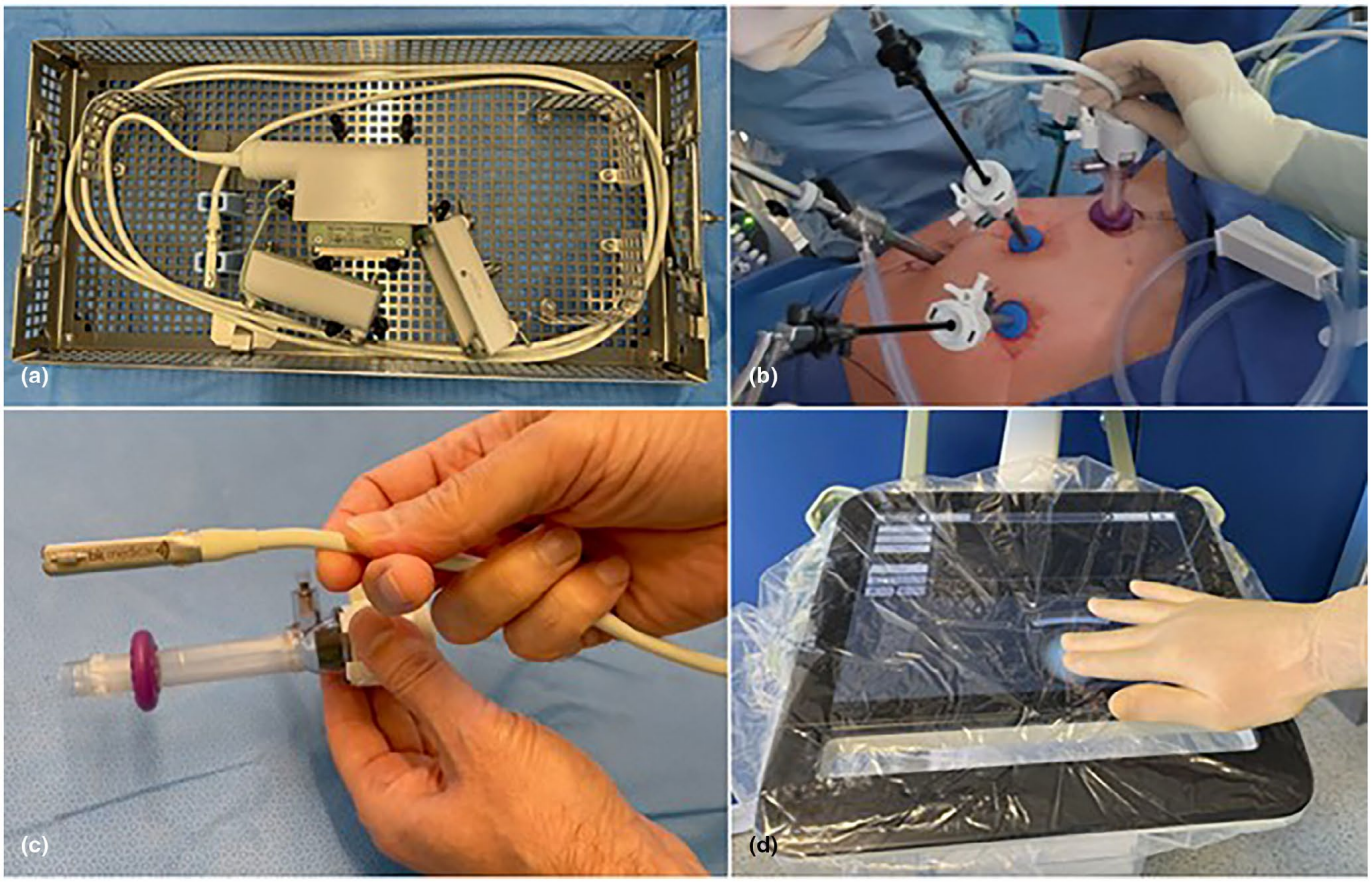
Setup for intraoperative ultrasound imaging. (a) Sterile drop‐in probe in the sieve tray. (b) Setting and application of the drop in probe through a 12 mm trocar suprapubically. (c) Tip of the drop‐in probe and the corresponding 12 mm trocar for application. (d) Sterile‐covered ultrasound control panel.

**FIGURE 2:**
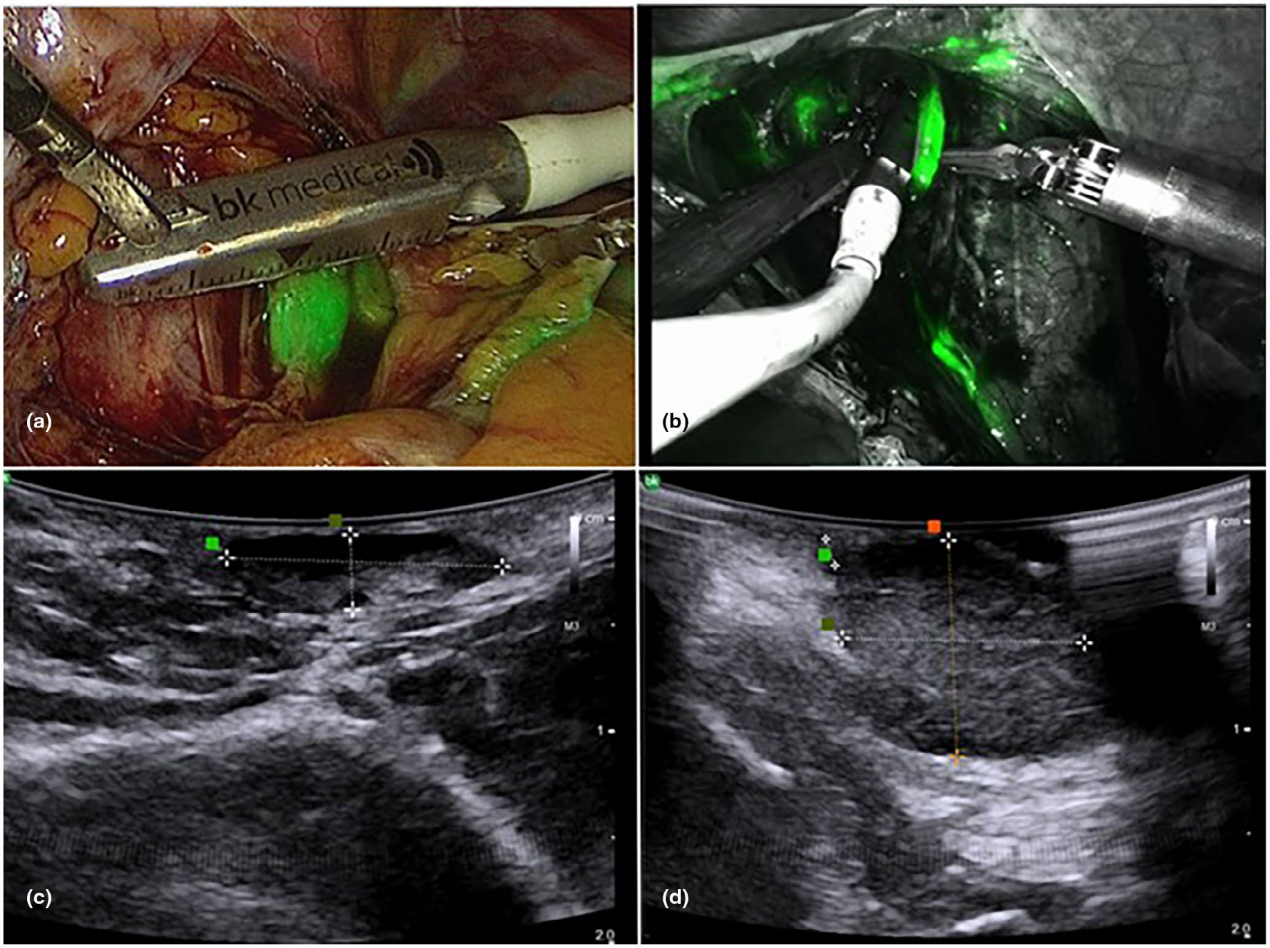
Intraoperative application of robotic ultrasound and representative findings. (a) Laparoscopic application of the ultrasound probe in indocyanine green (ICG) mode. (b) Robot assisted application of the ultrasound probe in ICG mode. (c) Image of a benign lymph node with long and short axis measurements. (d) Image of a malignant lymph node with long and short axis measurements.

Intraoperative sonographic evaluation of lymph nodes was successfully performed in all included patients (*n* = 20) and successfully demonstrated the feasibility and reliability of the technique in a clinical setting (Figures 1 and 2). No adverse events related to the sonographic procedure were reported.

### 3.2 Baseline demographics and characteristics

In the cohort of 20 patients, a total of 49 sentinel lymph nodes were detected using ICG imaging (Table 2). Patients presented with either cervical (*n* = 10, 50%) or endometrial cancers (*n* = 10, 50%). The patient cohort had a mean age of 50.2 years (range: 32.0–68.0 years). The mean body mass index (BMI, calculated as weight in kilograms divided by the square of height in meters) was 29.5 (range: 19.0–55.4). The mean duration of intra‐abdominal sonographic probe placement and measurement time was 6.50 min (range: 2.07–12.73 min). Laparoscopic surgery was found to be the predominant surgical approach (*n* = 15, 75%) followed by robot‐guided surgery (*n* = 5, 25%). Sentinel lymph node histopathology was negative (N0) in 85% of cases (*n* = 17), while 15% (*n* = 3) showed metastatic deposits, with no evidence of complications during or after the procedures.

**TABLE 2:** Patient characteristics.

IOUS of sentinel lymph node	Histopathology sentinel lymph node	Patient ID	Age	Type of tumor	Approach	BMI	Prior abdominal surgeries	Intra‐abdominal time of sonographic probe in minutes	FIGO‐classification	Histopathology T‐stadium	Histopathology N‐stadium
N‐	N‐	2	36	Cervical cancer	Laparoscopy	19.4	Yes	4.42	IB	pT1b1	N0
N‐	N‐	5	41	Cervical cancer	Laparoscopy	55.4	Yes	2.67	IA	pT1a	N0
N‐	N‐	7	59	Cervical cancer	Laparoscopy	22.84	Yes	9.85	IA	pT1a2	N0
N‐	N‐	10	43	Cervical cancer	Laparoscopy	21.3	No	4.82	IA	pT1a1	N0
N‐	N‐	14	54	Cervical cancer	Laparoscopy	21.3	No	8.08	IIIB	pT3b	N0
N‐	N‐	15	32	Cervical cancer	Laparoscopy	26.6	No	9.6	IIA	pT2a2	N0
N‐	N‐	20	40	Cervical cancer	Laparoscopy	29.4	No	8.88	IA	pT1a2	N0
N‐	N‐	1	68	Endometrial cancer	Laparoscopy	24.2	No	11.03	IA	pT1a	N0
N‐	N‐	8	59	Endometrial cancer	Laparoscopy	24.6	Yes	8.62	IA	pT1a	N0
N‐	N‐	11	61	Endometrial cancer	Laparoscopy	26.6	Yes	12.73	IA	pT1a	N0
N‐	N‐	13	53	Endometrial cancer	Laparoscopy	30.4	Yes	3.57	IA	pT1a	N0
N‐	N‐	16	51	Endometrial cancer	Laparoscopy	42.2	Yes	5.53	IA	pT1a	N0
N‐	N‐	19	67	Endometrial cancer	Laparoscopy	32.3	Yes	2.07	IA	pT1b	N0
N‐	N‐	4	65	Endometrial cancer	Robotical	22.1	No	10.13	IA	pT1a	N0
N‐	N‐	6	59	Endometrial cancer	Robotical	40.6	No	5.23	IA	pT1a	N0
N‐	N‐	12	41	Endometrial cancer	Robotical	28.4	Yes	5.1	IA	pT1a	N0
N‐	N‐	18	38	Endometrial cancer	Robotical	37.6	No	2.35	IA	pT1a	N0
N+	N+	3	61	Cervical cancer	Laparoscopy	37	No	4.15	IIA1	pT2a1	N1
N+	N+	9	33	Cervical cancer	Laparoscopy	19	No	4.83	IIIC1	pT3c	N1
N+	N+	17	44	Cervical cancer	Robotical	28.9	No	6.42	IA	pT1a1	N1

*Note*: BMI, calculated as weight in kilograms divided by the square of height in meters.

Abbreviations: BMI, body mass index; IOUS, intraoperative ultrasound.

A ROC analysis for the VITA/L/S score was performed to illustrate the relationship between specificity and sensitivity (Table 3). The AUC was 1.0. On this ROC curve, both sensitivity and specificity reached their maximum values of 1.0, which reflects ideal diagnostic performance. The Youden Index, which is calculated as sensitivity plus specificity minus one, also equaled 1 and confirmed the perfect balance between true positive and true negative rates.

**TABLE 3:** Contingency table for VITA/L/S score and histopathology of lymph nodes.

		Pathology		Total	Sensitivity	Specifity	Youden Index
		Benign	Malignant				
VITA/L/S score	0	40	0	40	1.0	1.0	1.0
	1	1	0	1			
	2	3	0	3			
	3	0	1	1			
	4	0	1	1			
	6	0	1	1			
	7	0	2	2			
Total		44	5	49			

Abbreviations: L/S, long‐to‐short axis; VITA, Vulvar International Tumor Analysis.

## 4 DISCUSSION

### 4.1 Summary of main results

The PelviSound proof‐of‐concept study successfully demonstrated the feasibility of intraoperative sonographic assessment of pelvic SLNs during laparoscopic and robotic surgeries for endometrial and cervical cancers. The study included 20 patients and yielded a total of 49 sentinel lymph nodes that were identified via ICG imaging. Intraoperative sonography was performed using a sterile drop‐in ultrasound probe and provided reliable imaging of lymph nodes with no reported adverse events. Notably, the sonographic assessment achieved a perfect sensitivity and specificity of 1.0 as confirmed by the area under the ROC curve (AUC = 1.0), indicating an ideal diagnostic performance.

### 4.2 Results in the context of what is known

SLNB has been widely adopted as a less invasive alternative to full pelvic lymphadenectomy in endometrial and cervical cancer management.^1^, ^3^, ^5^, ^6^, ^11^, ^12^, ^13^, ^14^, ^15^ The integration of ICG fluorescence has become the standard imaging technique and has led to further improvements in SLN detection rates.^4^, ^5^, ^16^, ^17^ However, intraoperative sonographic assessments of SLNs remain underexplored. While previous studies have primarily focused on preoperative imaging modalities such as magnetic resonance imaging (MRI), computed tomography (CT), and positron emission tomography (PET)‐CT for lymph node evaluation, recent advances demonstrate that ultrasound‐based techniques—such as quantitative ultrasound radiomics analysis and super‐resolution ultrasound localization microscopy of microvascular structure and flow—offer innovative and promising alternatives for assessing lymph node malignancy.^18^, ^19^, ^20^ The growing relevance of ultrasound in lymph node diagnostics is further underscored by the possibility of performing core needle biopsies of retroperitoneal lymph nodes under sonographic guidance, with contrast‐enhanced ultrasound (CEUS) representing another important innovation in this context.^21^

Our findings align with recent advancements in the application of ultrasound in gynecologic oncology. Fischerova et al. and Borges et al. highlighted the potential of ultrasound for lymph node staging, although these studies emphasized preoperative more than intraoperative use.^7^, ^8^ The present study bridges this gap and demonstrates that intraoperative ultrasound can offer real‐time, radiation‐free assessments with diagnostic accuracy that is comparable to histopathologic evaluation.

Intraoperative ultrasound could open new possibilities for risk stratification by allowing for more precise assessment of lymph node involvement during surgery. This real‐time imaging capability can help identify patients who may not require extensive surgical intervention, thus reducing the need for invasive procedures. By providing detailed visualization of SLNs and their characteristics, intraoperative ultrasound may support more tailored surgical approaches based on individual risk profiles. This approach could lead to a significant reduction in surgical morbidity while maintaining high diagnostic accuracy. Similar to the advancements obtained with artificial intelligence algorithms in intraoperative diagnostics, the use of intraoperative ultrasound offers a promising technique for optimizing surgical strategies and enhancing patient outcomes.

### 4.3 Strengths and limitations

This study presents several notable strengths. One of the most significant strengths is its innovative approach as it is the first study that explores the feasibility of intraoperative sonographic assessment of pelvic SLNs during laparoscopic and robotic surgeries for endometrial and cervical cancers. In this study, the method demonstrated exceptional diagnostic performance and achieved perfect sensitivity and specificity, which underscores its strong correlation with histopathologic findings. Additionally, the procedure's safety is well‐supported as no adverse events were reported throughout the study, indicating its suitability for clinical application. Another key advantage is the real‐time nature of the assessment that provides immediate feedback to surgeons during operations, which can enhance surgical decision making and potentially improve patient outcomes. Moreover, the technique is cost‐effective and radiation‐free as it leverages widely available ultrasound technology, which makes it accessible and beneficial in various healthcare settings, particularly in those with limited resources.

Despite these strengths, the study also has limitations that warrant consideration. Due to the nature of a proof‐of‐principal study, the relatively small sample size of 20 patients may limit the generalizability of the findings to broader populations, and larger studies are needed to validate the results. Since the study was conducted at a single center, it may also have been influenced by institutional biases that could have affected the external validity of the outcomes. Furthermore, the diagnostic accuracy of intraoperative sonography is highly dependent on operator expertise and experience; such dependence introduces potential variability in results across different practitioners. Beyond these general limitations, an important unresolved issue concerns the detection of low volume disease, including micrometastases and isolated tumor cells. Such lesions often induce only subtle or no morphological changes and therefore represent a diagnostic challenge for all imaging modalities, including ultrasound. Accordingly, the excellent diagnostic performance observed in this feasibility study should not be extrapolated to low volume metastatic disease. Future studies with larger cohorts and standardized ultrastaging protocols are required to specifically evaluate the sensitivity of intraoperative sonography for micrometastatic involvement and to better define its role in comprehensive nodal assessment.

### 4.4 Clinical and research implications

Clinically, the incorporation of intraoperative sonographic SLN assessment could enhance surgical decision making and potentially lead to a reduction in the need for extensive lymphadenectomy and its associated morbidities. This technique offers a cost‐effective, readily available imaging modality without radiation exposure, thus making it particularly advantageous in resource‐limited settings.

For research purposes, the study opens avenues for larger‐scale trials that could validate these findings and explore the integration of advanced sonographic techniques, such as elastography and contrast‐enhanced ultrasound, into intraoperative protocols. Future investigations should also assess the learning curve associated with this technique and its reproducibility across different surgical teams and institutions.

## 5 CONCLUSION

This study demonstrates that real time intraoperative sonographic assessment of pelvic SLNs during minimally invasive surgeries for endometrial and cervical cancers is feasible and safe, and shows promising diagnostic potential in a limited cohort. The technique offers high diagnostic accuracy without compromising patient safety, thus suggesting its potential as an adjunct to current SLNB protocols. However, due to the limited sample size, these findings require confirmation in larger, multicenter studies before clinical implementation can be recommended.

## AUTHOR CONTRIBUTIONS

Sascha Hoffmann conceived the study, oversaw its execution, and drafted the manuscript. Laura Trif was responsible for data collection and analysis. Bernhard Krämer, Felix Neis, Diethelm Wallwiener, Sara Y. Brucker, Markus Hoopmann, and Markus Hahn contributed to data acquisition and supervised the study. All authors critically revised the manuscript and approved the final version.

## FUNDING INFORMATION

The authors have nothing to report.

## CONFLICT OF INTEREST STATEMENT

The authors have no conflicts of interest.

## Data Availability

Data sharing is not applicable to this article as no new data were created or analyzed in this study.
